# Does Influencers Popularity Actually Matter? An Experimental Investigation of the Effect of Influencers on Body Satisfaction and Mood Among Young Chinese Females: The Case of RED (Xiaohongshu)

**DOI:** 10.3389/fpsyg.2021.756010

**Published:** 2021-11-26

**Authors:** Xiaoxiao Zhang, Wuchang Zhu, Shaojing Sun, Jingxi Chen

**Affiliations:** ^1^The School of Cultures, Languages and Area Study, University of Nottingham, Nottingham, United Kingdom; ^2^Department of Communication Studies, School of Languages and Communication Studies, Beijing Jiaotong University, Beijing, China; ^3^School of Journalism, Fudan University, Shanghai, China

**Keywords:** China, idealized images, body satisfaction, mood, social media influencer, self-discrepancy

## Abstract

Many studies have linked idealized body image on social media to negative psychological well-being among young females. However, social media influencers’ imagery has not attracted much research attention in either the Western or the Asian context. This study aimed to experimentally investigate the impact of high versus low popular social media influencer images on young Chinese females’ body satisfaction and mood. The participants were 420 female RED users (aged 18–35) who were randomly assigned to three groups: (1) the influencer-high group (idealized imagery alongside high engagement metrics); (2) the influencer-low group (the same idealized imagery adjusted for low engagement metrics); or (3) a control set of nature images. The results revealed that the groups exposed to influencer imagery had lower body satisfaction and more negative mood than the control group (nature images). Notably, this comparison showed no significant difference between the low-influencer and high-influencer groups in body satisfaction and mood. Additionally, this effect was moderated by individuals’ self-discrepancy between personal ideals and their own bodies. That is, exposure to idealized body images does not always produce harmful effects. For those with lower self-discrepancy, idealized body posts somewhat positively affected their body satisfaction. The current research contributes to the media effect literature by providing critical new insights into the study of body image in the context of China.

## Introduction

Influencers in Norway are now required by new regulations to label when their photos on social media have been retouched. The new regulations aim to fight the harmful effect of unrealistic beauty standards on the mental health of young Norwegians (Gerger, 2021). Currently, there is a general consensus that social media plays a dominant role in influencing norms and beauty standards about ideal body. Corresponding studies have detailed the detrimental influences on young females’ body satisfaction due to acute exposure to idealized images displayed on social media platforms (e.g., Fardouly et al., 2017; Hogue and Mills, 2019; Tiggemann and Anderberg, 2020; Chae, 2021). Social media influencers are “individuals who have built a sizeable social network of people following them and are seen as self-made micro-celebrities” (Shan et al., 2020, p. 591). They present idealized lifestyles by promoting material pursuit and vanity that speaks to the dreams of many ordinary people (Marwick, 2015). By creating attractive and high-quality content, influencers gain tens of thousands of followers and monetize their popularity through product promotion. Compared with traditional celebrities (e.g., movie/sports stars and singers), social media influencers are attractive to companies because they are perceived as authentic and trustworthy by social media users (Shan et al., 2020). Influencers also present an idealized physical appearance, with female influencers commonly adhering to the conventional Western female beauty norms of being young, trim, and feminine. For some young females, who are socialized to prioritize physical appearance, this curated imagery may contribute to the internalization of unrealistic appearance ideals, which can foster body dissatisfaction and adverse influence (Stice, 2001).

Globally, most body image studies have concentrated on Facebook and Instagram, mainly in the Western context (e.g., Tiggemann and Slater, 2013; Meier and Gray, 2014). However, limited research has investigated Chinese social media platforms. China, as the world’s largest social network market, has the most dynamic environment for social media (Chiu et al., 2012). Today, social media has become an integral part of young Chinese people’s daily lives, and it is not easy to find someone who is not using it. Facebook and Instagram are widely used in Western society, but they have been blocked in the Chinese market. As a result, a domestic Chinese Internet services company has developed an alternative: Little Red Book (小红书, also known as RED). RED is a content-sharing site where users can post text, images, and short videos in the form of notes and can like, comment, or forward posts to their friends through links. RED was established in 2014 and attracted more than 300 million registers by July 2019. The most active user group was dominated by the post-90s generation (born after 1990). RED generates three billion note exposures per day, 70% of which are derived from user-generated content (Bray and Lee, 2021). As one of the most popular social media platforms, RED has attracted many active young female influencers. They show off their privileged lives while presenting smooth, flawless skin, perfect faces, and trim bodies, attracting the gaze of viewers. Young female influencers tend to upload only their “best” personal photos, which are carefully chosen to maximize their attractiveness beauty and are then augmented by filters and digital alteration. When viewing the influencer-generated content, other users come to formulate a view of what is normal, acceptable, or unacceptable in terms of appearance and body shape in their community. The end is that comparisons with female influencers on RED are most often upward in direction, resulting in dissatisfaction with users’ own body and appearance. For this reason, RED has been receiving increasing complaints and criticism from the public for disseminating distorted beauty standard (Yip and Ding, 2021).

To date, few studies have been conducted specifically on social media influencers’ imagery and its impact on viewers. However, previous studies have confirmed that attractive celebrities’ images reinforce the unattainable ideal of thinness, resulting in upward social comparison and body dissatisfaction among young viewers. For instance, Shorter et al. (2008) found that young females’ favorite celebrities become unrealistic targets for their upward social comparison. Individuals who think that their body image are quite different from their favorite celebrities are prone to disordered eating attitudes and behaviors. Similarly, Harrison (1997) found that the interpersonal attractiveness of thin celebrities seems to be positively correlated with people’s eating disorder symptoms compared with ordinary or heavy celebrities. Brown and Tiggemann (2016) explored the impact of Instagram photos of celebrities and attractive peers on young females’ emotions and body dissatisfaction. The results demonstrated that experimental groups exposed to images of celebrities and peers had more negative emotions and body dissatisfaction than the control group (nature images), but there was no significant difference between famous celebrity images and attractive peer images.

Engagement is the key for social media influencers to gauge their success and business value (Cotter, 2019). Often, the metric is an index of engagement that helps describe influencers’ popularity rankings. For example, according to Ismail (2018), those with more than 1M followers and more than 100k followers are considered mega-influencers and macro-influencers, while those with only 1k to 100K followers are micro-influencers. Similarly, De Veirman et al. (2017) believes that the number of followers can easily determine the popularity of social media influencers, while others believe that the number of likes can properly reflect the popularity of online celebrities (Reich et al., 2012; Hong and Cameron, 2018; Kim and Xu, 2019). As such, social media users are likely to encounter idealized and staged influencer imagery accompanied by a wide range of metrics. Among other studies, the impact of viewing metrics has been examined among young adult audiences. Sherman et al. (2018) found that when viewing Instagram users’ photos with more likes (vs. a few likes), young audiences experience greater brain activity, suggesting that they scan well-liked photos more thoroughly than less favored photos (Sherman et al., 2018). In this way, it is possible that high engagement metrics may work analogously to the warning tags on edited social media images, turning the viewer’s attention to the perfection of influencers’ body shape and the perceived relative shortcomings of their own body image. Therefore, this study views engagement metrics as a key indicator of influencer popularity status. Furthermore, we will also assess whether the level of self-discrepancy moderates the relation between idealized influencer imagery and body satisfaction, as many studies suggest that the impact of body image exposure on one’s body satisfaction may depend upon the level of self-discrepancy one holds. (e.g., Bessenoff, 2006; Knobloch-Westerwick and Romero, 2011; Yu et al., 2013; Ahadzadeh et al., 2017). Therefore, the following hypotheses are proposed:

Hypothesis 1 (H1): Viewing an influencer’s body images along with both high and low engagement metrics results in greater negative mood and lower body satisfaction than viewing the control images of nature.
Hypothesis 2 (H2): Viewing an influencer’s body images along with high engagement metrics results in greater negative mood and lower body satisfaction than viewing the same images along with low popularity metrics.
Hypothesis 3 (H3): Self-discrepancy will moderate the association between influencers’ idealized body images and young females’ body satisfaction.

To address these gaps, this study investigates the effect of idealized body imagery of Chinese influencers with different popularity (high vs. low) on female social media users’ body satisfaction and mood and whether their self-discrepancy moderates this effect. To our knowledge, this is the first study to test how influencers’ body imagery affects individuals’ perception and evaluation of their body image. Additionally, this is one of the few explorations examining self-discrepancy as a moderator for the impact of influencers’ body imagery and body satisfaction of young female users. Thus, this study provides contributions to the media effect literature by offering critical understandings and new insights into the study of body image in the context of China.

## Methods

### Participants and Procedure

Female participants (*N* = 420) were recruited through a famous Chinese data company between May 10, 2021 and May 19, 2021. The participants’ ages ranged from 18 to 35 (*Mage* = 27.95; *SD* = 6.49). They had a mean body mass index (BMI) of 19.37 (*SD* = 2.29). A total of 77.38% of participants were normal weight, followed by overweight (15.71%), underweight (3.61%) and obese (3.25%), based on the widely used Chinese BMI categories suggested by the National Health Commission of China (2013) (BMI < 18.5: underweight, 18.5 ≤ BMI < 24.0: normal weight, 24.0 ≤ BMI < 28.0: overweight and BMI ≥ 28.0: obese).

The study was approved by the research ethics committee of Beijing Jiaotong University. Because this was an online experiment, participants logged in anonymously to a survey website provided by the data company through their mobile phones and watched the experimental stimuli. After giving informed consent, they were randomly assigned to one of three conditions: (1) influencers with high engagement metrics; (2) influencers with low engagement metrics; or (3) control (natural environment images). Participants first completed measures of body satisfaction, mood, and self-discrepancy. Next, they were shown a series of RED influencers’ posts (a profile page and a set of body images).

Each participant viewed a slideshow of 3 influencers’ profile pages and 24 body images on their own mobile phone. Each image was displayed for at least 15 s, during which the participants could not skip the image and go to the next page. Additionally, to ensure that participants viewed the images, they responded along dimensions including quality, appropriateness, interestingness, and enjoyableness. After the images were shown, the participants again reported their mood and body satisfaction and finally answered questions about their demographics.

### Experimental Stimuli

Influencer images: The stimulus materials were designed to resemble a typical RED influencer’s post to replicate naturalistic mobile-based social media engagement. Three female influencers from RED were chosen. We deliberately did not select well-known and easily recognizable influencers to prevent participants from being influenced by previous impressions. For each influencer, we selected their profile page and a post including 8 body images. Each profile page was edited to the same background color and size, and the profile picture was a clear selfie. The influencer’s name, gender, and RED ID were displayed, while the rest of the information on the profile page, including location, education, bio and user level, was blurred out to reduce visual interference. The images depicted the influencers in artistic, lifestyle, and travel selfies. They were fully clothed in summer attire, and most photos appeared to be staged or posed rather than spontaneous. For highly popular influencers, the engagement metrics on the profile page were manipulated to over 1M followers and over 1M likes and saves. The engagement metrics of each post were manipulated to over 3k likes, 1k comments and 1k saves^1^. For low-popular influencers, the same profile pages and images were used with the number of followers and the number of likes and saves adjusted for low popularity, defined for this purpose as influencers with fewer than 1k followers, fewer than 100 likes and saves and fewer than 10 comments on each post. This classification is based on the annual report of the Little Red Book (Guang, 2020) and previous literature on the classification of influencers (Ismail, 2018; Turner, 2020).

Nature images: Regarding the control (natural environment) images, three RED accounts that posted nature photos were chosen. Participants viewed the 3 profile pages and 24 photos depicting natural objects and scenery (i.e., a path in a green forest). Both the profile pages and the nature photos were given lower popularity metrics, separating the effects that occurred as a product of viewing idealized female bodies versus nature imagery from the effects of seeing popularity metrics.

### Measures

#### Mood and Body Satisfaction

In this study, a visual analogue scale (VAS) was used to measure five mood dimensions, anxiety, depression, happiness, anger and self-confidence, and two body satisfaction dimensions, weight satisfaction and appearance satisfaction. Participants indicated their current feelings by moving a slider on a horizontal line with endpoints marked “None” to “Very Many,” capturing responses ranging from 1 to 100. The scores for happiness and confidence were reversed, and the individual dimensions were averaged to provide general scores of body satisfaction (*M* = 56.79, *SD* = 15.04) and mood (*M* = 58.51, *SD* = 14.98).

#### Self-Discrepancy

Self-discrepancy was evaluated by the Body-Image Ideals Questionnaire (BIQ) (Szymanski and Cash, 1995). In this study, body proportions and body weights were evaluated. The scale has two components, perceived discrepancy, and perceived importance of the ideal, both of which are used for the final construct of the cross-product, weighted difference. Participants were asked to think about their personal ideals (What kind of person do you want to be) and to rate on a 4-point scale to measure how much their own body matched the ideal one (Cash et al., 2004; Ahadzadeh et al., 2017). Discrepancy was rated on a scale ranging from −1 (exactly the same as me) to +3 (very unlike me), and the importance was rated on a range from 0 (unimportant) to 3 (very important). The weighted difference score was the mean of two (Discrepancy × Importance) cross-products. According to Szymanski and Cash (1995), it is crucial to assign 0 to an insignificant ideal, which “results in a cross-product of 0, properly discounting the extent of discrepancy,” while assigning −1 to any matched (non-discrepant) ideal “creates continuity of the Weighted Discrepancy score from very important self-ideal congruities to very important discrepancies” (p. 469). The final measurement of discrepancy in terms of body weight (*M* = 1.99, *SD* = 1.57) and body proportion (*M* = 1.69, *SD* = 1.61) was moderately correlated, *R* = 0.78, *p* < 0.001, *R* = 0.634, *p* < 0.001, and thus averaged (*M* = 1.84, *SD* = 1.36).

#### Body Mass Index

Participants were asked to report their height and weight in centimeters and kilograms, which was used to calculate the body mass index (BMI) (*M* = 19.37, *SD* = 2.29).

### Statistical Analysis

All data collected in this study were analyzed using SPSS 27.0. Pearson correlations were conducted to test the bivariate associations among age, BMI, body satisfaction (pre- and postexposure), and mood (pre- and postexposure). One-way analysis of variance (ANOVA) was used for the randomization check and manipulation check. To test our hypotheses, separate one-way analyses of covariance (ANCOVA) with BMI, age and pre-exposure body satisfaction as covariates were conducted. Additionally, *post hoc* analyses using Tukey’s HSD (honest significant difference) were conducted to compare the nature images control group with a combination of the two influencer groups and then compare the highly popular and low popular images to each other. We then tested a moderation model using Hayes (2018) PROCESS Model 1. The bootstrapping method was used to test the mediation effects. This method produced 95% bias-corrected confidence intervals of the estimates from 5,000 resamples of the data. For a multi-categorical independent variable, we used indicator coding to produce two dummy variables, and the control condition (nature images) was used as a reference group.

## Results

Preliminary analyses indicated that the experimental conditions had no significant difference with respect to age, *F*(2,417) = 0.19, *p* = 0.83; BMI, *F*(2,417) = 9.31, *p* = 0.11 and there was no significant difference in pre-exposure body satisfaction, *F*(2,417) = 0.7, *p* = 0.49 and pre-mood, *F*(2,417) = 0.53, *p* = 0.58. For the manipulation check for influencers’ engagement metrics, participants (*N* = 80) indicated whether they believe that the stimuli depicted a RED influencer. One-way ANOVA revealed significant differences between conditions, *F*(2,239) = 282.97, *p* < 0.001. A Tukey *post hoc* test (all *p* < 0.001) showed that highly popular influencer profiles were significantly more commonly identified as “influencers” (*M* = 5.38, *SD* = 0.74), followed by less popular influencer profiles (*M* = 3.77, *SD* = 0.69) and then nature controls (*M* = 2.80, *SD* = 0.73). All descriptive statistics and correlation matrices for all the variables are provided in Table 1.

**TABLE 1:** Descriptive statistics and Pearson correlation for the key variables.

Variables	*M* (*SD*)	1	2	3	4	5	6
1. Age	28.33 (3.96)	–					
2. BMI	19.37 (2.29)	0.13**	–				
3. Body satisfaction (pre-exposure)	55.25 (14.75)	0.18**	0.13**	–			
4. Mood (pre-exposure)	59.00 (14.28)	0.12*	0.14**	0.39**	–		
5. Body satisfaction (post-exposure)	56.58 (15.32)	0.18**	0.04	0.81**	0.28**	–	
6. Mood (post-exposure)	58.03 (15.68)	0.13**	0.06	0.23**	0.78**	0.42**	–

N = 420; M, mean; SD, standard deviation; BMI, body mass index. *p < 0.05; **p < 0.01.

**FIGURE 1:**
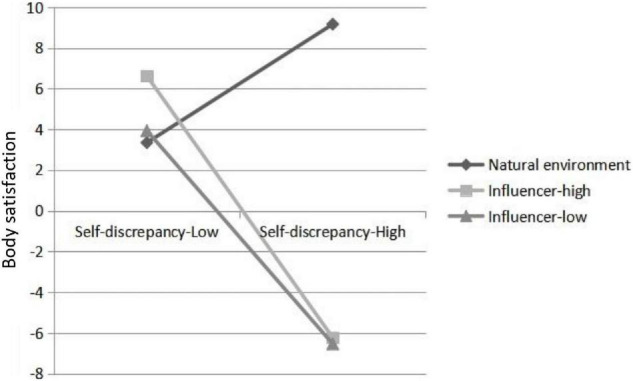
Interaction effects of image types and self-discrepancy on body satisfaction. Error bars represent 95% confidence interval.

Our first two hypotheses predicted the direct impact of RED influencers’ body posts and different levels of engagement metrics on other RED users’ mood and body satisfaction. The results of H1 showed that image type had a significant impact on post-exposure body satisfaction and post-exposure mood, *F*(2,414) = 9.73, *p* = 0.01; *F*(2,414) = 4.54, *p* = 0.01. The first planned comparison showed that viewing the high- and low-popularity images led to lower body satisfaction and greater negative mood than viewing the nature control images, *t*(418) = 2.93, *p* = 0.004, *t*(418) = 2.97, *p* < 0.001. Therefore, H1 was supported. The result of H2 revealed that there was no significant difference between the image group with high popularity and the image group with low popularity in terms of young females’ body satisfaction and mood, t(277) = 1.09, *p* = 0.28; *t*(277) = 0.55, *p* = 0.13. H2 was not supported. For H3, the interaction between influencers’ idealized body images (both high and low popularity) and females’ self-discrepancy was found to be statistically significant, β = −9.34, *p* < 0.001; β = −8.15, *p* < 0.001. The analysis results of the moderation model are presented in Table 2. The conditional effect of influencers’ body images on females’ body satisfaction showed corresponding results. Specifically, when a participant’s self-discrepancy was low, there was a significant positive relationship between influencer image groups (both high and low popularity) and body satisfaction, β = 6.61 *p* < 0.001; β = 3.47, *p* < 0.05. However, when a participant’s self-discrepancy was high, there was a significant negative relationship between influencer imagery groups (both high and low popularity) and young females’ body satisfaction, β = −18.72, *p* < 0.001; β = −18.60, *p* < 0.001. To better understand the conditioning effect, estimated values of body satisfaction are plotted in Figure 1. The results show that the relationship between idealized body images and body satisfaction varies with the degree of an individual’s self-discrepancy.

**TABLE 2:** The results of moderation analysis.

				95% confidence interval		
	
	Effect	β	*SE*	Lower	Upper	*t*
**Outcome: Body satisfaction (post-exposure)**						
Imagery groups 1	1-0	–6.06	1.79	–9.578	–2.53	−3.38***
Imagery groups 2	2-0	–7.56	1.8	–11.101	–4.03	−4.21***
Self-discrepancy	Self-discrepancy	2.91	1.09	0.771	5.04	2.68**
Imagery groups 1 × Self-discrepancy	1-0 × Self-discrepancy	–9.34	1.4	–12.099	–6.59	−6.67***
Imagery groups 2 × Self-discrepancy	2-0 × Self-discrepancy	–8.15	1.36	–10.82	–5.47	−5.99***
*R* ^2^	0.23***					
*F*	24.92					

**p < 0.01; ***p < 0.001.

## Discussion and Conclusion

The primary aims of the current study were to examine the effect of popularity metrics accompanying RED influencers’ body images on young Chinese females’ mood and body satisfaction and to further investigate the moderating effect of their self-discrepancy on this effect. In so doing, this study has added new insights and expanded the current research on the detrimental effects of social media on body image by highlighting the role played by influencers imagery. First, as predicted, idealized influencers’ imagery causes lower body satisfaction and more negative mood compared to natural environment images. However, there were no differences between influencers’ body images with high compared to low engagement metrics. Second, self-discrepancy was found to moderate the effect of exposure to influencers’ images on young females’ body satisfaction.

The findings related to the first two hypotheses underscore the detrimental psychological impacts of idealized social media imagery reported in the existing literature (e.g., Brichacek et al., 2018; Tamplin et al., 2018; Sherlock and Wagstaff, 2019). Specifically, acute exposure to thin and attractive female influencer imagery had an immediate negative impact on young Chinese females’ mood and body satisfaction. Interestingly, exposure to influencer images with both high and low metrics had a similar detrimental impact, which echoes the finding of Tiggemann et al. (2018), who suggested that thin-ideal body posts on social media caused lower body satisfaction among young females regardless of the number of likes accompanying body images. Similarly, our experimental results revealed that even if the engagement metrics (e.g., followers, likes, and saves) are clearly indicated, individuals are nonetheless affected by the content of the posts, not the engagement metrics below the body photos. This is contrary to Chua and Chang’s (2016) conclusion that teenage girls reported they were more attracted to peers’ photos on social media with a higher number of “likes” and were more likely to make upward comparisons with their peers’ photos with a higher number of likes. It should also be recognized that the photos displayed in the present study belonged to attractive influencers, while in Chua and Chang’s study, girls were required to respond to known peers in their social network. It is possible that “likes” are especially meaningful to teenage girls, who attach great importance to their peers’ opinions. However, our research found that “likes” are no longer significant among adult women because the average age of our research sample was 28 years old. A similar result was found in the study of Osburn (2005), which found that peer comments attached to photographs were associated with body dissatisfaction for girls between the ages of 14 and 16. However, another age group, i.e., women aged from 25 to 35, was not found to be negatively affected.

The findings of the present study can be used to guide daily social media usage. However, the comparably adverse influences for images across levels of popularity make defining practical guidelines for safe social media use complicated. Simply avoiding idealized body pictures from a particular group of users is insufficient because idealized images may come from social media influencers with different levels of popularity and even ordinary users. Like Instagram and RED, social media encourages all users to modify their photos to be ideal. Our findings suggest that when photos are edited to be equally idealized on social media platforms, popularity may not matter. Given this finding, we suggest that China, like Norway, also introduce laws related to social media posts that require all content creators to disclose when they have retouched or added a filter to photos.

Although influencers’ engagement metrics were irrelevant here, the present study nevertheless showed that favored influencers can affect people’s body image attitudes. In our experiment, participants reported lower body satisfaction and higher negative mood after viewing RED influencers’ body photos compared to those who viewed the nature environment photos. Currently, female influencers dominate social media and they have increasing become key opinion leaders in Chinese society. They arguably represent ideal beauty in social media and cultural beauty ideals. As current beauty ideals emphasize thinness for women, it is suggested that this constant presentation of edited female body images in social media reinforces an unattainable thin ideal and causes the prevalence of body dissatisfaction among young women (Maltby et al., 2005; Brown and Tiggemann, 2016). Given that the strong influence of female influencers, they can be encouraged to upload real images for other users to view, as previous finding revealed that exposure to the social media influencers’ real images resulted in a decrease in body dissatisfaction, relative to exposure to the their ideal images (Tiggemann and Anderberg, 2020).

Additionally, we noticed a slight increase in body satisfaction after participants viewed control images portraying a natural environment. This finding has been confirmed by previous literature (e.g., Hennigan, 2010; Swami et al., 2016, 2018). Numerous studies have indicated that humans have an innate preference for natural settings and that interacting with nature can positively influence people’s health and well-being (Grinde and Patil, 2009). For example, literature reviews and meta-analyses have consistently noted that being in a natural setting is related to an improvement in psychological well-being, including health improvement, higher self-esteem, healthier emotions, and more physical energy (Grinde and Patil, 2009; Abraham et al., 2010; Bowler et al., 2010; Russell et al., 2013; Sandifer et al., 2015). In addition to psychological health in general, some scholars believe that spending time in a natural environment improves self-perceptions of body image and reduces body image concerns. This is mainly because the natural environment helps “limit negative appearance-related thoughts, restrict the influence of internalized negative appearance-based stereotypes, and promote speedier recovery from threats to body image” (Swami et al., 2018). Time spent in the natural environment also separates the individual, to a certain extent, from the social environment that values appearance (Hennigan, 2010), which in turn nurtures the development of the ability to critically evaluate the appearance of ideal images and engage in the behavior of protecting the body (Swami et al., 2016).

Notably, this study extends previous research about media effects on body image by elaborating the mechanisms underlying social comparison processes. The moderating effect of self-discrepancy contributes to a more nuanced understanding of body image processes. It is often assumed in existing research and widespread belief that idealized thin body imagery inevitably has a negative influence on females’ body satisfaction (e.g., Hogue and Mills, 2019; Tiggemann and Anderberg, 2020). Our findings challenge this hypothesis and show that exposure to idealized body imagery is not always harmful and may even be beneficial, especially for people with low self-discrepancy. Our results partially support the findings of previous studies (e.g., Lockwood and Kunda, 1997; Knobloch-Westerwick, 2015; Veldhuis et al., 2017); that is, posting idealized body photos on social media does not always have a negative impact. Specifically, our results reveal that this negative effect does exist, with one difference: for individuals who have a lower discrepancy between their personal ideal and self, viewing idealized body images does not always negatively impact their body satisfaction. This can be explained by self-determination theory, which underlines people’s motivations, goals, and aspirations for perceptions, cognitions, and other related well-being. Namely, exposure to idealized body images may enhance the motivation for self-enhancement more prominently, especially among people with low self-discrepancy. However, those with high self-discrepancy may not be motivated because of the large differences and gaps that already exist. When the portrayed idealized body is considered to be achievable, exposure to idealized images can stimulate motivation for self-improvement rather than self-evaluation (Deci and Ryan, 2012). Therefore, for individuals with low self-discrepancy, the idealized body image may look closer to or like their bodies, thus generating greater body satisfaction through self-enhancement motivation. In contrast, for individuals with high self-discrepancy who are dissatisfied with their own bodies, idealized body posts may aggravate the original differences and lead to lower body satisfaction.

## Limitations and Implications

This study has a few limitations. First, we focused only on young women’s psychological response to idealized images of social media influencers. Young males were not included in our sample, which may affect the generalization of the results. Future research should consider the similar negative impact of idealized social media images on young males. Second, the average age of our sample was relatively high, i.e., 28 years old, which may further influence the results. Third, as mentioned in the previous section, the number of likes on photos posted on social media is more meaningful for adolescent girls, while adult women seem to be less susceptible to engagement metrics below the postings. In addition, online experiments may affect participants’ attention, although we tried to avoid any such effect through quality control.

## Data Availability Statement

The original contributions presented in the study are included in the article/supplementary material, further inquiries can be directed to the corresponding author.

## Ethics Statement

The studies involving human participants were reviewed and approved by Beijing Jiaotong University, School of Language and Communication Studies, Ethic Research Committee. The patients/participants provided their written informed consent to participate in this study.

## Author Contributions

XZ designed the study, conceived the analysis question, conducted the analysis, drafted the manuscript, and approved the final version. JC and SS critically revised the manuscript content. WZ was responsible for organizing experiments and collecting questionnaires. All authors have read and agreed to the published version of the manuscript.

## Conflict of Interest

The authors declare that the research was conducted in the absence of any commercial or financial relationships that could be construed as a potential conflict of interest.

## Publisher’s Note

All claims expressed in this article are solely those of the authors and do not necessarily represent those of their affiliated organizations, or those of the publisher, the editors and the reviewers. Any product that may be evaluated in this article, or claim that may be made by its manufacturer, is not guaranteed or endorsed by the publisher.

## Footnotes

1 On the profile page of RED, the number of likes and the number of saves are counted together.
