# Corrigendum: Does Influencers Popularity Actually Matter? An Experimental Investigation of the Effect of Influencers on Body Satisfaction and Mood Among Young Chinese Females: The Case of RED (Xiaohongshu)

**DOI:** 10.3389/fpsyg.2022.873514

**Published:** 2022-03-09

**Authors:** Xiaoxiao Zhang, Wuchang Zhu, Shaojing Sun, Jingxi Chen

**Affiliations:** ^1^The School of Cultures, Languages and Area Study, University of Nottingham, Nottingham, United Kingdom; ^2^Department of Communication Studies, School of Languages and Communication Studies, Beijing Jiaotong University, Beijing, China; ^3^School of Journalism, Fudan University, Shanghai, China

**Keywords:** China, idealized images, body satisfaction, mood, social media influencer, self-discrepancy

In the published article, there was an error in affiliation 1. Instead of “Faculty of Social Sciences, School of Sociology and Social Policy, University of Nottingham, Nottingham, United Kingdom”, it should be “The School of Cultures, Languages and Area Study, University of Nottingham, Nottingham, United Kingdom.”

The authors apologize for this error and state that this does not change the scientific conclusions of the article in any way. The original article has been updated.

## Publisher's Note

All claims expressed in this article are solely those of the authors and do not necessarily represent those of their affiliated organizations, or those of the publisher, the editors and the reviewers. Any product that may be evaluated in this article, or claim that may be made by its manufacturer, is not guaranteed or endorsed by the publisher.

